# Late Prevalence of Typical and Atypical Symptoms of Frey’s Syndrome after Parotidectomy for Benign Tumor

**DOI:** 10.3390/jpm14010096

**Published:** 2024-01-16

**Authors:** Maria Raffaella Marchese, Federica Rossi, Stefano Settimi, Jacopo Galli

**Affiliations:** 1 Otolaryngology Division, Department of Neurosciences, Sensory Organs and Thorax, Fondazione Policlinico Universitario “A. Gemelli” IRCCS, 00168 Rome, Italy; mariaraffaella.marchese@policlinicogemelli.it (M.R.M.); jacopo.galli@unicatt.it (J.G.); 2 Section of Otolaryngology, Head-Neck and Sense Organs Department, Catholic University of the Sacred Heart, 00168 Rome, Italy; federica.rossi01@icatt.it

**Keywords:** Frey’s syndrome, parotidectomy, itch, flushing, gustatory sweating

## Abstract

Background: The treatment of choice for tumors located in the parotid gland is surgery. Nevertheless, postoperative complications are not infrequent. Regardless of the type of surgical procedure, the most common complication is Frey’s syndrome (FS). Traditionally, FS includes unilateral gustatory sweating and flushing of the facial skin lining the parotid compartment. Recent research describes atypical discomfort associated with FS. The aim of this study was to assess the late prevalence and severity of both usual and atypical symptoms after parotidectomy for benign tumors. Methods: We conducted a cross-sectional study involving 86 subjects who underwent superficial parotidectomy at least one year before the study. The questionnaire included the sweating–flushing–itch–paresthesia–pain (SFIPP) Frey scale supplemented by specific questions about symptoms. Results: Sixty-seven out of eighty-six (77.9%) cases reported almost one symptom. The most frequent symptom was itch (36/67—53.7%), followed by pain (35/67—52.2%), while 28/67 (41.8%) subjects complained of atypical symptoms without flushing or sweating. A desire to treat the discomfort was reported by 50/67 (74.6%) subjects. Conclusions: Late postparotidectomy local discomfort is not infrequent and includes both usual and “unusual” symptoms almost equally. Our results suggest the importance of informing patients about the occurrence of the syndrome and the available treatment options during pre- and postoperative counseling.

## 1. Introduction

Tumors of the salivary glands constitute 3% of all head and neck tumors. Of these, parotid gland (PG) tumors have been at the center of scientific attention in recent decades because PG is the most common site, involved in 85% of cases. About 80% of them are of benign origin and are most frequently located within the superficial lobe of the parotid gland [1]. Pleomorphic adenomas (PAs) are commonly reported as the most diagnosed histologic subtype, more frequently affecting women, while Warthin’s tumors (WTs) are the second most common histotype, with a documented increase in incidence rate over the last few years [2]. The size of PG tumors ranges from a few millimeters to several centimeters, with an average of 2–6 cm. Nonetheless, PG tumors can grow to be quite large if left untreated due to their sneaky growth and lack of symptoms. The treatment of parotid gland tumors is eminently surgical, and the technique has undergone constant development in recent decades in order to minimize postoperative complications. Complications following parotid gland surgery can be divided into early complications, mid-term complications, and late complications depending on when they occur. Early complications include temporary or permanent paralysis of the facial nerve and capsular tears. Complications that occur between 7 and 20 days postoperatively are defined as medium-term complications and are salivary fistulas and sialoceles. Finally, complications that occur more than 20 days after the operation are long-term consequences and include Frey’s syndrome (FS), abnormal scarring, first bite syndrome, wound complications, and recurrences [3]. The onset of the different complications after parotid gland surgery is related to the surgery performed. As recently described by Committeri et al. (2023) [4], regardless of surgical procedure, the most frequent complication is FS, followed in descending order by temporary facial paralysis, wound dehiscence, salivary fistula, pathological scarring, permanent facial paralysis, and recurrence.

“Auriculotemporal syndrome”, better known as “Frey’s syndrome”, is described as sweating and/or flushing occurring in the auriculotemporal nerve region almost immediately after tasting [5]. The most common cause of Frey’s syndrome is salivary gland surgery. Larger parotid tumors are associated with more extensive dissection and parasympathetic nerve fiber exposure and damage, resulting in a higher risk of Frey’s syndrome [6]. Aside from parotidectomy, FS may also occur after other types of neck surgery, facelifts, infections, or trauma to the parotid region [7], in people with diabetes mellitus without apparent triggers, or in pediatric patients, mostly those with a history of forceps births [8]. Bilateral symptoms are more likely to be idiopathic. Familial bilateral FS has been described in patients without a history of trauma, leading to the hypothesis that congenital dysfunction of the auriculotemporal nerve is the cause [9].

Frey L. hypothesized a pathological mechanism based on an aberrant reinnervation of the facial sweat glands by surgically interrupted parasympathetic secretomotor fibers directed to the parotid gland [10,11]. Because aberrant nerve regeneration takes time to produce gustatory sweating, the presentation of FS is generally delayed by 6 to 18 months after surgery [9]. The result is a local vasodilatation (gustatory flushing) and localized sweating (gustatory sweating) of the skin in response to mastication and salivation. In the literature, Redleaf and McCabe [12] described the histopathologic features of skin affected by FS. They found hyperplasia and hypertrophy of sweat glands, probably in response to aberrant parasympathetic influence.

Symptoms tend to occur during meals, especially when spicy or sour food is being consumed. Another theory for the development of FS is that damaged sympathetic nerve fibers lead to increased sensitivity of the sweat glands, which can then be stimulated by acetylcholine released from neighboring parasympathetic fibers. This theory may be convincing in cases where gustatory sweating occurs shortly after surgery, when nerve regeneration has not yet taken place.

In rare cases, gustatory sweating may occur in areas distant from the parotid gland and not directly involved by surgery [13], as reported by Caliò et al. [14], who described a case of excessive gustatory sweating on the forehead and scalp resulting from iatrogenic damage during maxillofacial and temporomandibular joint surgeries, indicating complex nerve interactions. Another distal site described as affected by FS after parotidectomy is the temporal region. This likely develops either by the regeneration of severed postganglionic fibers into sympathetic targets distally along the course of the auriculotemporal nerve or by their regeneration into fibers of the sympathetic plexus traveling along the superficial temporal artery.

The incidence of Frey’s syndrome has been reported as highly variable, ranging from 17% to 100% of patients who undergo parotidectomy [7,15]. This imprecise result is due both to the non-systematic diagnosis and to the underestimation of this syndrome in some studies. In fact, most authors in the past investigated Frey’s syndrome based on patients’ self-reported subjective complaints, without standardized questionnaires. Recently, a specific test, named the Minor starch–iodine (MSI) test, was deemed the gold standard for the diagnosis and measurement of the severity of Frey’s syndrome [16]. The MSI is based on the chromatic effect that results from the interaction of sweat with iodine and starch. In addition to providing objective evidence of abnormal reinnervation, the test is particularly useful for determining the exact extent of the affected skin area, planning local treatment, and recording the affected areas for comparison with the results of a follow-up examination. Nevertheless, the MSI is not a quick test. It takes at least 20 min, as it is a multi-stage test that includes a preparation phase, stimulation phase, observation, and final cleansing. The correlation between subjective symptoms and the results of objective measurement (MSI test) is still to be clarified.

In recent years, more attention has been paid to the prevention of FS. The most important method to prevent the development of FS is creating a barrier at the time of surgery that prevents defective nerve regeneration between the parasympathetic secretory fibers and the sweat glands in the parotid region. Several techniques are described in the literature. All are associated with risks and disadvantages, including the creation of a donor site, prolonged surgical time, variable efficacy in preventing FS, wound infection, rejection, and postoperative complications. Prevention techniques include a thick skin flap, acellular dermal matrix, autologous fat implantation, superficial muscular aponeurotic system (SMAS) flap, temporoparietal fascia (TPF) flap, and sternocleidomastoid muscle (SCM) flap. In this regard, Roh et al. [17] demonstrated that the incidence of Frey’s syndrome may be reduced with the closure of the exposed parotid parenchyma and that covering it with fascia is preferred over non-closure to prevent Frey’s syndrome.

Among recent findings, the importance of other associated symptoms burdening the quality of life of patients submitted to parotidectomy has emerged as a new topic to pay attention to. In fact, besides classical symptoms, which are always present but with a variable severity, Frey’s syndrome can be also associated with other frequent complaints, such as pain, itch, and paresthesia in the auriculotemporal area. A systemic, comprehensive evaluation of Frey-related complaints is lacking. Most studies describe a single or predominant manifestation and measure subjective outcomes simply by the presence or absence of symptoms, without grading them. Anecdotally, the Frey Questionnaire Card has been used in the literature [18]. It is borrowed from a chart used in general medical practice to define and measure the patient’s functional status. In order to assess the severity of symptoms attributable to iatrogenic FS, we specifically designed and adopted an illustrated visual analogue scale called “Sweating-Flushing-Itch-Paresthesia-Pain Frey” (SFIPP-Frey scale), which includes the typical and atypical symptoms of FS [19]. Moreover, its utility was also demonstrated in the assessment of the results obtained with the injection of botulinum toxin A (BoNT-A), a proven effective treatment for different combinations of symptoms [19]. Nevertheless, a systematic and comprehensive assessment of these complaints has been poorly described in the literature. The aim of this work was to assess the late prevalence and severity of both usual and atypical postparotidectomy symptoms and to evaluate the associations between them.

## 2. Materials and Methods

### 2.1. Study Design and Population

A survey with a cross-sectional study design was conducted in April 2022 at the Operative Unit of Otorhinolaryngology of the Fondazione Policlinico Universitario A. Gemelli IRCCS, Rome, Italy, in patients who had undergone superficial parotidectomy for benign parotid tumors at least one year before, between January 2015 and February 2021. The surgical technique did not change during the study period. Exclusion criteria were facial palsy of any grade; history of neurologic disease or diabetes; other types of parotidectomy or parotidectomies for malignant disease (due to frequent need for adjuvant treatments); previous radiant treatment of the head and neck area; age < 18 or >75; insufficient data collected.

### 2.2. Data Collection

Data collection was based on an internet survey conducted through Google Forms (Google LLC, Mountain View, CA, USA). We emailed the link to the form to all the enrolled patients, asking them to fill in the 12 items with the required information. Items 1 to 8 were based on the SFIPP-Frey scale [19], which measures the severity of gustatory sweating (S), gustatory flushing (F), itch (I), paresthesia (P) (abnormal sensation of the skin with no apparent physical cause), and pain (P) (score range for each symptom: 0 (absence of discomfort)–4 (severe discomfort)). The total score of the SFIPP-Frey scale was obtained by adding the scores for each item (range 0–20). Items regarding itch, paresthesia, and pain were supplemented by questions about the triggering condition (during meals or regardless of meal). Finally, the questionnaire included a question with multiple-choice answers about the site of affected area (preauricular, retroauricolar, temporoparietal, cheek, retro-angulomandibular, and forehead) and a question about the global severity of symptoms (“mild”, “moderate”, “almost severe”, and “severe”), their evolution over time, and the presence of ongoing treatments or wish for treatment. Moreover, we recorded the type of parotidectomy incision for each patient: modified Blair incision and facelift incision. A modified Blair incision combines an inverted L-shaped (hockey stick) preauricular Blair incision with a cervical limb extending into the neck. Its advantages are the exposure of the entire periphery of the gland and excellent access to the facial nerve. It raises a robust flap that resists flap necrosis. The incision further allows for extension into a neck dissection incision and cervicofacial flap elevation. It is cosmetically acceptable and if placed in a natural skin crease, it is difficult to discern.

A facelift incision originates at the superior root of the helix and lies just inside the anterior edge of the tragus, curving superiorly around the lobule towards the mastoid, preserving the sulcus between the lobule and the cheek, and continues in the postauricular crease to the occipital hairline without traversing the hairless mastoid region, and then extends approximately 6 cm downward to the edge of the hairline. A large flap of skin is elevated, limiting anterior exposure and access to the neck for dissection. This incision is ideal for benign, posteriorly located tumors.

Written informed consent was obtained from all patients enrolled in this study. The protocol of this study was reviewed and approved by the Institutional Review Board of the “Fondazione Policlinico Universitario A. Gemelli IRCCS”, Catholic University of the Sacred Heart (ID 3494—prot. n. 0043203/20).

### 2.3. Statistical Analysis

Data were exported to Excel sheets. Statistical analysis was performed using commercially available software (version 2019, Excel; Microsoft Corp.; Redmond, WA, USA). Continuous variables were described using means and standard deviations (SDs) when their distribution was normal and frequencies and percentages when their distribution was skewed.

Comparison between groups were performed using a *t*-test for paired samples in normally distributed values. Normality was checked with a graphical test Q-Q plot. The significance level was set at 0.05, with a confidence interval of 95%.

## 3. Results

From a series of 122 patients with surgical history of superficial parotidectomy for benign parotid tumors, 7 subjects did not answer the survey (5.73%). Out of the 122 patients, 29 (29.5%) met the exclusion criteria and 86/122 (70.5%) patients were considered eligible. We excluded 11/86 patients (12.7%) because of insufficient data, 9/36 (25%) due to diabetes, 8/36 (22.2%) due to undergoing parotidectomy for malignant disease, and 1/36 (2.7%) was excluded due to being affected by neurologic disease.

Out of the remaining patients, 37/86 (43%) were males and 49/86 (57%) were females, with a mean age of 51.1 years (SD ± 11). The mean time between the surgery and the survey was 16.7 months (SD ± 7). Sixty-seven patients reported at least one symptom (77.9%) (Sympt group) and nineteen were asymptomatic (22.1%) (Asympt group). The demographic data of each group are shown in Table 1. There was no statistically significant difference between the time from parotidectomy to survey calculated in each group (Sympt group, 18.1 months vs. Asympt group, 17.2 months; *p* > 0.05).

**Figure 1:**
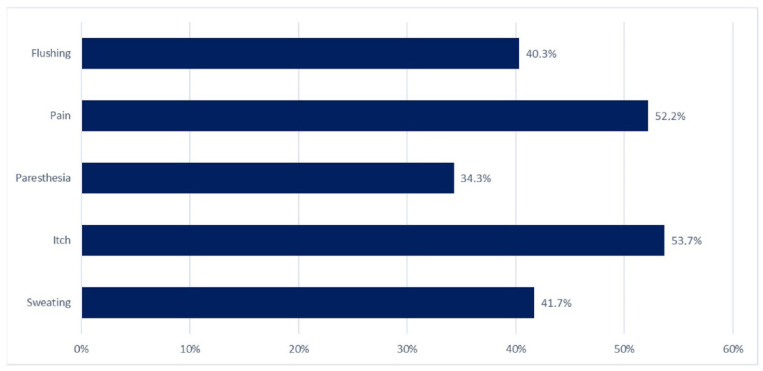
Prevalence (%) of the symptoms (flushing, pain, paresthesia, itch, and sweating).

Figure 1 shows the prevalence of each symptom in the Sympt group. The most frequent one was itch (36/67—53.7%), followed by pain (35/67—52.2%). In most cases, itch or pain occurred regardless of meal consumption (94.4% and 71.4%, respectively). Out of the 67 symptomatic cases, 39 (58.2%) reported flushing (27/39—40.3%) or sweating (28/39—41.7%). Among the symptomatic patients, 28/67 (41.8%) subjects complained of atypical symptoms without flushing or sweating.

**Figure 2:**
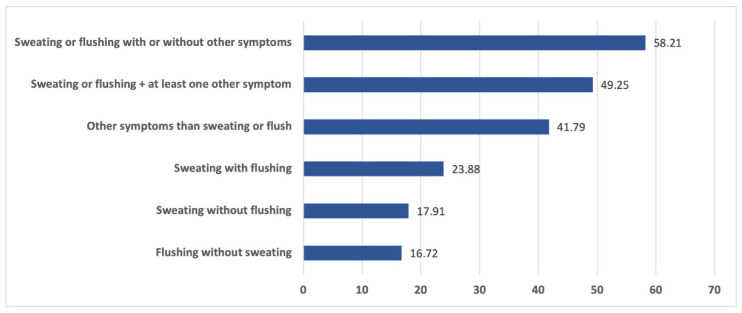
Prevalence (%) of flushing and sweating and their association with atypical symptoms.

Figure 2 shows the prevalence of flushing and sweating and their association with other symptoms. Six out of sixty-seven patients (8.9%) complained of all the symptoms under investigation. The most frequent association of symptoms was itch with paresthesia (23/67—34.3%). Sixty out of sixty-seven subjects (89.5%) experienced a mild or moderate overall severity of symptoms. More specifically, 19/67 (28.4%) cases experienced “moderate” severity and 2/67 (2.9%) experienced “almost severe” severity. None reported an overall “severe” rating of the symptoms.

Based on total SFIPP-Frey scores, symptomatic patients were distributed as follows: 1–5 score in 48/67 (71.6%) cases, score ranging from 6 to 10 in 17/67 (25.4%), 11–15 score in 2/67 (2.9%), and 16–20 score in 0/67 (0%) cases. The affected areas, in decreasing order, were retromandibular (39/67—58.2%), preauricolar (34/67—50.75%), cheek (27/67—40.3%), retroarticular (22/67—32.8%), temporal (3/67—4.48%), and forehead (2/67—2.9%). Symptoms had worsened over time in 4/67 (5.9%), improved in 46/67 (68.7%), and were unchanged in 17/67 (25.4%) of patients. At the time of the survey, 5/67 (7.5%) subjects were in therapy for FS and 50/67 (74.6%) declared a wish to start therapy to reduce all discomfort.

Finally, according to the type of surgical incision (modified Blair incision versus facelift incision), we did not find statistically significant differences in terms of the presence of symptoms, SFIPP score, symptom severity, and tumor volume (*p* > 0.05) (Table 2).

## 4. Discussion

Our results demonstrated that, at least one year after surgery, nearly 80% of patients who had undergone superficial parotidectomy complained of at least one symptom in the parotid region. More than half of the cases suffered from gustatory flushing or sweating, suggesting FS. In the literature, the incidence of FS after parotidectomy varies widely (range 10–98%) depending on the type of surgery and on both time point and method of assessment [2]. It is known that FS may be underdiagnosed in short-term analyses because this condition generally develops 6–18 months after surgery [10]. Our incidence was similar to that obtained by Neumann et al. [10] (62.2%) in a long-term follow-up. Nevertheless, the literature reports a number of patients who underwent parotidectomy and have a positive Minor starch–iodine test result which is higher than their self-reported incidence of symptoms [10], suggesting the “subclinical” nature of FS [20]. In this regard, we believe that it is important to distinguish between self-reported, surveyed, and tested FS. Our overall prevalence, obtained by specific questions and consistent with that reported by Koch et al. [21], is higher than the self-reported and lower than the objective prevalence described in the literature [22]. Therefore, we suggest that patients be routinely asked specific questions about discomfort in the parotid compartment during postoperative consultations.

Interestingly, 42.8% of cases with sweating did not also complain of flushing. In agreement with our result, Tugnoli et al. [23] demonstrated that in FS, sweating is not invariably accompanied by gustatory erythema. On the other hand, we detected patients with flushing and without gustatory sweating in 40.7% of cases. Both typical symptoms are subsequent to the reinnervation of blood vessels and sweat glands, respectively, through the miscommunication of parasympathetic auriculotemporal nerve fibers [24]. Probably, as argued by Tugnoli et al. [23], this difference in prevalence is due to the variable concentration of sweat glands and blood vessels in the affected skin. Flushing is not caused by substances deriving from pathological gland activation but from afferent nociceptive pathway activation, induced by mastication.

The high prevalence (about 50%) of the association between the typical symptoms of FS and at least one atypical discomfort, most frequently itch and pain, is worth noting. These last data support the result we first described in a previous study [19]. In addition, our survey evidences the high prevalence (40%) of symptomatic subjects who complained of atypical discomforts in the absence of sweating and flushing, mostly of itch and pain. The superior root of the auriculotemporal nerve contains general somatic afferent fibers that provide sensation to the external ear, posterior temporomandibular joint, and the temple. Because of its complex path, it is easily exposed to surgical damage, thus causing pain or paresthesia in all terminal branches distal to the lesion.

The overall severity of Frey’s syndrome was mild or moderate. The results of the self-scored severity scale (five-point Likert scale) were in line with the SFIPP-Frey scores. Finally, the absence of differences in terms of the presence of symptoms, SFIPP score, and symptom severity according to the type of surgical incision (modified Blair incision versus facelift incision) suggests a minor impact of surgical approaches on the prevalence of usual and atypical postparotidectomy symptoms, thus confirming that the aberrant reinnervation of the facial sweat glands may take place regardless of the type of skin incision.

Frey’s syndrome is often regarded as a minor complication and its significance for patients is frequently underestimated by surgeons. When questioned, many patients do not recall being adequately informed about the risk of developing Frey’s syndrome after parotid surgery. Preoperative counseling and education about Frey’s syndrome are critical as part of the informed consent process to ensure timely diagnosis, initiate appropriate treatment, and meet patients’ perioperative expectations. Postoperative symptoms such as facial warmth, facial flushing, and sweating associated with acidic or spicy foods should be communicated to the operating surgeon.

The social impact of Frey’s syndrome should not be ignored. In a questionnaire for patients who had undergone parotidectomy for benign salivary disease, gustatory sweating was cited as the most serious and worrying complication. Patients reported a reduced quality of life, difficulty enjoying meals, and a general malaise that worsened over time. In order to alleviate and address the accompanying social impact, early patient education and prompt diagnosis and treatment are essential.

In most of the cases, the syndrome improved over time and in about one in four subjects, the overall discomfort did not change. However, the syndrome can result in discomfort as well as social anxiety and avoidance [20]. A survey conducted by Baek and colleagues [24] revealed that FS was the most common self-perceived consequence of parotidectomy for benign disease with significant psychosocial impact [25]. A great proportion of our subjects (75%) declared a wish to undergo therapy. This result indirectly confirmed the morbidity of postsurgical parotid region discomfort, contrasting with its definition as a “subclinical” disorder. Interventions to treat these consequences have focused the attention of researchers in the last years.

In addition to surgical measures, such as transection of the auriculotemporal nerve, Jacobson’s neurectomy, excision of the affected skin, or the interposition of fascia lata, muscle flaps (platysma), or silastic sheeting (temporary), there are also drug treatments, consisting of the injection of alcohol into the otic ganglion, systemic or topical application of anticholinergics, antihydrotics, or antiperspirant [22,26]. Nevertheless, nowadays, the risks related to the surgical options are not justified for a benign entity such as FS. Surgery is reserved for refractory cases where conservative or medical therapies are no longer effective.

On the other hand, it has been demonstrated that topical antiperspirants cannot totally stop gustatory perspiration: less than 50% of patients report any advantages and they last for less than a day [27]. Moreover, the injection of alcohol into the otic ganglion has been used in the past, but it can lead to anesthesia of the mandibular branch of the trigeminal nerve, which can cause even more troublesome symptoms than Frey’s syndrome [28]. Systemic atropine is not effective in preventing sweating; if anhidrosis occurs with its use, this is usually a sign of atropine overdose, which can also have other adverse side effects such as tachycardia, blurred vision, disorientation, respiratory distress, and coma [28].

Outside these options, topic BoNT-A injection is a safe and effective treatment choice for Frey’s syndrome [29,30]. BoNT-A acts as an anticholinergic and blocks the release of acetylcholine at the neuromuscular junction by degrading synaptosomal-associated protein 25 (SNAP-25) [31]. The injection causes the chemical denervation and paralysis of both striated muscles and sweat glands [32]. Peak effects occur within 4 to 7 days, and patients report an improvement in gustatory sweating, facial flushing, and overall quality of life [25]. However, chemical denervation diminishes over time with absorption of the denatured SNAP-25 and restoration of the relationship between the nerve ending and the lamina terminalis [31]. Therefore, repeat injections are often required as symptoms may recur in 27% and 92% of patients after 1 and 3 years, respectively [33]. Several study groups have demonstrated a significant decrease in the affected sweating area and a progressively lower need for toxin dosage after repeated injections with BoNT [22]. We previously demonstrated for the first time the efficacy of BoNT-A to reduce FS and associated atypical symptoms [19]. Nevertheless, only a very small percentage of the studied population was already in treatment for the reported symptoms, while most wished to start treatment.

In conclusion, it is of paramount importance to make patients aware during preoperative counselling of the high risk of Frey’s syndrome after surgery. Similarly, it is important to ask patients about local complaints during follow-up in order to detect discomfort and to propose suitable treatment options.

## Figures and Tables

**Table 1:** Demographic data of the study groups.

	Sympt	Asympt
Number of subjects	67/86 (78%)	19/86 (22%)
Sex	28 M; 39 F	9 M; 10 F
Mean age (years)	49.64	57.36
Average of months between surgery and survey	18.1 (SD ± 9) *	17.2 (SD ± 8) *

Sympt: symptomatic group; Asympt: asymptomatic group. * Sympt vs. Asympt difference: *p* > 0.05.

**Table 2:** Comparison of results according to the type of surgical incision.

	Modified Blair Incision (n = 76)	Facelift Incision (n = 10)
Presence of symptoms (cases)	59/76 (77.6%)	8/10 (80%)
SFIPP score (mean)	4.1	5.5
Symptom severity (median)	1	1
Tumor volume (mean; cm^3^)	34.8	29.1

## Data Availability

The data presented in this study are available on request from the corresponding author. The data are not publicly available due to privacy restrictions.
